# A convex 3D deconvolution algorithm for low photon count fluorescence imaging

**DOI:** 10.1038/s41598-018-29768-x

**Published:** 2018-07-31

**Authors:** Hayato Ikoma, Michael Broxton, Takamasa Kudo, Gordon Wetzstein

**Affiliations:** 1 Stanford University, Department of Electrical Engineering, Stanford, 94305 United States; 2 Stanford University, Department of Chemical and Systems Biology, Stanford, 94305 United States

## Abstract

Deconvolution is widely used to improve the contrast and clarity of a 3D focal stack collected using a fluorescence microscope. But despite being extensively studied, deconvolution algorithms can introduce reconstruction artifacts when their underlying noise models or priors are violated, such as when imaging biological specimens at extremely low light levels. In this paper we propose a deconvolution method specifically designed for 3D fluorescence imaging of biological samples in the low-light regime. Our method utilizes a mixed Poisson-Gaussian model of photon shot noise and camera read noise, which are both present in low light imaging. We formulate a convex loss function and solve the resulting optimization problem using the alternating direction method of multipliers algorithm. Among several possible regularization strategies, we show that a Hessian-based regularizer is most effective for describing locally smooth features present in biological specimens. Our algorithm also estimates noise parameters on-the-fly, thereby eliminating a manual calibration step required by most deconvolution software. We demonstrate our algorithm on simulated images and experimentally-captured images with peak intensities of tens of photoelectrons per voxel. We also demonstrate its performance for live cell imaging, showing its applicability as a tool for biological research.

## Introduction

Widefield deconvolution microscopy is a classical technique to computationally enhance the contrast of images captured with a widefield fluorescence microscope^1–5^. While this technique has been widely used to visualize biological structures^6–8^, it remains difficult to apply in practice. Accurate results require correct modeling of the microscope’s point spread function (PSF) and careful manual calibration of camera gain and noise characteristics. This places a significant burden on experimentalists, as they must thoroughly measure characteristics of their microscope in order to achieve results that are free from bias or reconstruction artifacts.

Successful application of deconvolution also requires knowledge of the properties of the deconvolution algorithms themselves. Particularly important are the subtle modeling assumptions each algorithm makes about image noise, boundary conditions, and signal priors. Incorrect assumptions result in a variety of reconstruction artifacts including the amplification of image noise, ringing artifacts around objects, or even realistic looking but incorrect image features that can be misleading and difficult for the human eye to detect. Noise generally causes these artifacts to become more pronounced. Such artifacts are particularly likely to occur when imaging in the low photon count regime (e.g., with an average of less than 100 photons per pixel), such as the light levels required to minimize phototoxicity and bleaching during live cell imaging. Therefore, successful application of deconvolution to very low signal-to-noise ratio (SNR) images requires considerable expertise in image reconstruction and it is considered an active area of research^9^.

In this paper we propose a new 3D deconvolution algorithm designed for imaging biological specimens at very low light levels. Central to our approach is a noise model that accounts for both photon shot noise and camera read noise using a mixed Poisson-Gaussian noise model. We have formulated a convex loss function using the noise model, which we minimize using a problem splitting framework and the alternating direction method of multipliers (ADMM) algorithm^10,11^. Because the loss function is convex, this algorithm is guaranteed to converge to its global minimum. Additionally, by using techniques from image processing, our algorithm also estimates noise parameters and camera gain values on-the-fly directly from the captured focal-stack image^12–14^. This eliminates the need to hand-tune noise parameters or separately perform noise estimation from calibration images.

To further improve reconstruction quality in the presence of very high noise levels, we incorporate a structural prior: the Hessian Schatten-norm regularizer^15^. This regularizer is an extension of the total-variation (TV) norm, but whereas the TV norm tends to produce piecewise constant images^16^ (i.e., “staircase” artifacts when applied to biological images), this Hessian regularizer promotes piecewise-smoothness and allows continuous changes in intensity across structures. It is therefore proposed to be more suitable for biological imaging applications^17,18^. Additionally it holds desired properties such as convexity, contrast covariance, rotation invariance, translation invariance and scale invariance.

Finally, our algorithm reduces ringing artifacts. Most deconvolution algorithms utilize the fast Fourier transform (FFT) to compute a convolutional image formation model. As a result, images are incorrectly modeled to be periodic both laterally and axially. This periodic assumption produces discontinuities at the image borders resulting in ghosting and ringing in restored images^7,19^. In the early deconvolution literature, it was common practice to avoid this issue by capturing a focal stack whose edges are completely dark (i.e., one that entirely contains the microscopic object in the focal stack). However, this again places a burden on the experimentalist, who must modify experimental protocols and collect additional z-stack images in order to achieve acceptable deconvolution results. Apodization and padding are often applied as a pre-processing step to address these artifacts^7^, but these techniques inherently alter the measurement and can introduce error into the reconstructed volume. Rather than alter the measurement, our approach is to extend the boundaries of the reconstructed volume beyond that of the focal stack. Sometimes called the “undetermined boundary condition,” this helps to move modeling errors away from biological structures at the center of the volume and has been shown to perform better than apodization or padding^19^.

In the next section we describe our deconvolution algorithm in detail. Then, we use simulated images to show that our choice of the mixed Poisson-Gaussian noise model and the Hessian regularizer reduces the error in 3D reconstruction as compared to other modeling assumptions commonly used in deconvolution algorithms. Next, the performance of the proposed algorithm is experimentally validated using fluorescent beads and fixed cellular samples. In these experiments, we compare our results to those produced by popular commercial and non-commercial deconvolution software. To assess the accuracy of each technique, we quantitatively compare deconvolved data to volumes captured using confocal microscopy. Finally, we present results from a live cell imaging experiment to show the practicability of our method.

## Results

In widefield fluorescence microscopy, volumetric data is collected by capturing multiple images with different focus settings. These images form a three dimensional array, or “focal stack,” that serves as the input for a deconvolution algorithm. The algorithm also relies on an accurate PSF model for the microscope, a noise model, and regularization that introduces prior information about the structure of the object being reconstructed. In this section, we show our mathematical formulation for these models and describe how to implement our 3D deconvolution algorithm. We then validate its performance on experimentally captured fluorescent beads and cells.

### Point spread function model

An accurate model of a microscope’s PSF is essential for correct deconvolution. We use Debye’s model to accurately simulate this PSF for a high numerical aperture (N.A.) microscope objective^20^. However, it is computationally expensive to simulate a 3D Debye PSF, as this requires numerical integration at each point of a 3D sampling grid. Therefore, we simulate the amplitude PSF (APSF) (i.e., the complex-valued, scalar wavefront at the image plane) based on the Debye’s model for a single point centered on the optical axis on the focal plane of the microscope. Then, we take the Fourier transform of this “in-focus” APSF to compute the amplitude transfer function (ATF) of the microscope. A defocused version of the APSF can then be efficiently computed by multiplying the ATF by an appropriate defocus factor, which will be described below, followed by an inverse Fourier transform to produce a defocused APSF. The intensity PSF of the microscope is then computed by taking the squared modulus of the APSF. This process is commonly referred to as forming the generalized aperture model of the microscope^21,22^.

We now describe this process in more detail. A typical widefield fluorescence optical microscope has a circular aperture, and we assume that the microscope’s PSF is depth-invariant. This assumption is accurate when performing deconvolution on thin biological samples, as we do in this work. For such a microscope, the Debye’s APSF model at the focal plane is

1 $${h}_{{\mathscr{A}}}(x,y)\,:=\,\frac{T}{{f}_{{\rm{o}}{\rm{b}}{\rm{j}}}^{2}{\lambda }^{2}}{\int }_{0}^{\alpha }P(\varphi ){J}_{0}(2\pi k\,\sin (\varphi )\sqrt{{x}^{2}+{y}^{2}})\sin (\varphi )\,d\varphi ,$$

where $(x,y)\in {{\mathbb{R}}}^{2}$ represents the coordinates on the image sensor, *f*_obj_ is the focal length of the objective lens, *T* is the magnification factor of the imaging system, $\alpha \,:={\sin }^{-1}({\rm{NA}}/n)$ is the half-angle of the N.A., $k\,:=n/\lambda$ is the wave number, *J*_0_(·) represents the zeroth-order Bessel function of the first kind, and $P(\varphi )\,:\,=\,\sqrt{cos(\varphi )}$ is the design condition for the Abbe-sine corrected objective lens^20^. The N.A. of the objective lens, the refractive index of the immersion medium and the emission wavelength are denoted by NA, *n* and *λ*, respectively. By computing the numerical integration of the model (1) over a 2D sampling grid, we can obtain the APSF for a point on the optical axis at the focal plane.

The ATF corresponding to this APSF can be computed by back-propagating the APSF from the sensor plane to the aperture plane through the tube lens. It is well known that wave propagation from the image plane to the back aperture plane of the microscope can be described as the Fourier transform with a coordinate scaling factor, and we denote the in-focus ATF by $H(u,v)\,:\,={ {\mathcal F} }^{-1}\{{h}_{{\mathscr{A}}}(x,y)\}(u,v)$, where $(u,v)\in {{\mathbb{R}}}^{2}$ are the coordinates on the aperture plane.

Finally, we multiply the microscope ATF by the appropriate phase delay corresponding to defocus by *z* relative to the nominal object plane. Then we propagate the wavefront back to the image plane via another Fourier transform. This results in the defocused PSF,

2 $$h(x,y,z)={|{\mathscr{F}}\{H(u,v)D(u,v,z)\}(x,y)|}^{2},$$

where $D(u,v,z)\,:\,=\,\exp \{\,-\,i2\pi z\sqrt{{k}^{2}-{({\rm{N}}{\rm{A}}/\lambda )}^{2}({u}^{2}+{v}^{2})}\}$ is the defocus factor. This “defocus ATF,” introduced by Hanser *et al*., does not make use of the paraxial approximation^23^, and thus this approach can be used to model a high N.A. objective lens. To reflect the effect of a pixel size in our simulation, the PSF is evaluated over a 4× finer sampling grid and averaged to match the area of an image sensor pixel. The definition of the transform ${\mathcal F}$ is given in Supplementary Section A.

### Image formation and noise model

Scientific digital cameras are typically photon shot noise and read noise limited. Therefore, as pixels independently measure a moderate light exposure, the overall noise at each pixel can be modeled as a mixture of signal-dependent Poisson noise and the signal-independent Gaussian noise^24,25^. With this noise model the signal at the *j*-th voxel of the focal stack, which is represented as a vector ${\bf{y}}\in {{\mathbb{R}}}^{M}$, is written as

3 $${{\bf{y}}}_{j}=\gamma {\mathscr{P}}(({\boldsymbol{Ax}}{)}_{j})+{\mathscr{N}}\mathrm{(0,}{\sigma }^{2}),$$

where *γ* is the camera gain of the imaging system; i.e., the conversion factor from photoelectrons to digital units. ${\mathscr{P}}(\theta )$ represents a realization of the Poisson noise with the rate $\theta \in {{\mathbb{R}}}_{+}$, and ${\mathscr{N}}\mathrm{(0,}{\sigma }^{2})$ denotes a realization of the Gaussian noise that models read noise with zero mean and a variance *σ*^2^. The subscript *j* denotes the *j*-th element of its corresponding vector. The matrix ${\boldsymbol{A}}\in {{\mathbb{R}}}^{N\times N}$ is the linear image formation operator representing the three-dimensional convolution with the microscope’s PSF. The signal ${\boldsymbol{x}}\in {{\mathbb{R}}}^{N}$ represents the volume to be reconstructed measured in units of photoelectrons over a time frame corresponding to the exposure time of each captured image. This is achieved by normalizing the sum of the PSF *h* to be one in each *xy*-slice (see detailed discussion in Supplementary Section H).

Read noise is typically very small (1–2 photoelectrons per pixel is typical of modern sCMOS imagers). As a result, if there are roughly 20 or more detected photons per pixel, the dominant source of noise will be photon shot noise, and in all but the most photon-starved applications read noise can be safely ignored. This is why many deconvolution algorithms rely solely on Poisson statistics. However, for images with very low photon counts or images with some bright pixels and many very dark pixels (which is common is fluorescence imaging), the read noise is not negligible. This is why we have adopted a mixed Poisson-Gaussian noise model.

The model presented here does not include the camera offset as it can be simply subtracted from captured images with the dark-field correction. Additionally, we model the camera gain *γ* to be uniform over the image sensor. This assumption is reasonable for CCD cameras but the gain varies from pixel to pixel in CMOS cameras due to the electronic architecture^25^. Therefore, we suppress this sensitivity variation using flat-field correction, which we described as a pre-processing step (see Methods). After applying the dark-field correction and the flat-field correction, the image is divided by the camera gain to obtain a signal that follows the Poisson-Gaussian noise model: ${\tilde{{\bf{y}}}}_{j}={\mathscr{P}}(({\boldsymbol{Ax}}{)}_{j})+{\mathscr{N}}\mathrm{(0,}\,{\tilde{\sigma }}^{2})$ where ${\tilde{{\bf{y}}}}_{i}\,:\,={{\bf{y}}}_{i}/\gamma$ and $\tilde{\sigma }\,:\,=\sigma /\gamma$. As the probability density function of the mixed Poisson-Gaussian random measurements is a convolution of the Gaussian and Poisson distributions, its log-likelihood function involves an infinite sum and cannot be evaluated in closed form. Therefore, we approximate it using the shifted Poisson noise model^26–29^:

4 $${\tilde{{\bf{y}}}}_{j}\approx {\mathscr{P}}(({\boldsymbol{Ax}}{)}_{j}+{\tilde{\sigma }}^{2})-{\tilde{\sigma }}^{2}\mathrm{.}$$

The shifted Poisson probability distribution has the same mean and variance as the original Poisson-Gaussian noise model, and this holds true for any photon count. In addition, the negative log-likelihood function for the shifted Poisson model has a closed form that is analytically tractable, and it is therefore straight-forward to derive its proximal operator (see Supplementary Section E) in our optimization framework.

When we apply the proposed noise model (4), the camera gain *γ* and the read noise variance *σ*^2^ have to be known prior to performing deconvolution. Traditionally, *σ*^2^ is estimated from a dark image by taking its sample variance, and *γ* is estimated from a set of images of autofluorescence plastic slides or fluorescence solutions captured with different exposure times^25,30^. These are time consuming calibration steps that are not always well understood by practicing microscopists. We prefer to avoid this pre-estimation operation altogether by estimating *γ* and *σ*^2^ directly from the measured focal stack data itself. For this purpose, we use Foi’s noise estimation algorithm before performing deconvolution^12^. Foi’s method consists of two steps: (1) accumulate noise mean-variance pairs estimated locally over an image, and (2) perform a maximum likelihood fitting of the mean-variance pairs to the noise model. This method can handle saturated pixels and is known to be robust, and in Supplementary Section F we have further assessed the performance of this estimator on experimentally-captured fluorescence images.

### Deconvolution using ADMM

Our deconvolution algorithm minimizes a penalty function that is defined as the sum of a data fidelity term $\ell (\,\cdot \,)\,:{{\mathbb{R}}}^{N}\to {\mathbb{R}}$, a regularization term ${\mathcal R} (\,\cdot \,)\,:{{\mathbb{R}}}^{N}\to {\mathbb{R}}$, and a non-negativity constraint. The data fidelity term $\ell$ utilizes the negative log-likelihood function based on the shifted Poisson noise model described in (4), and the regularizer ${\mathcal R}$ is chosen to be the Frobenius norm of the Hessian of an image, which is one of the Schatten-Hessian norms^17,18^:

5 $$\mathop{{\rm{m}}{\rm{i}}{\rm{n}}{\rm{i}}{\rm{m}}{\rm{i}}{\rm{z}}{\rm{e}}}\limits_{{\boldsymbol{x}}}\,\ell ({\boldsymbol{x}})+\nu {\mathscr{R}}({\boldsymbol{x}})+{{\mathscr{I}}}_{[0,+{\rm{\infty }})}({\boldsymbol{x}})\,{\rm{w}}{\rm{i}}{\rm{t}}{\rm{h}}\{\begin{array}{c}\ell ({\boldsymbol{x}})\,:\,={\sum }_{j\in {\mathscr{C}}}(({\boldsymbol{A}}{\boldsymbol{x}}{)}_{j}-({\mathop{{\bf{y}}}\limits^{ \sim }}_{j}+{\mathop{\sigma }\limits^{ \sim }}^{2}){\rm{l}}{\rm{o}}{\rm{g}}(({\boldsymbol{A}}{\boldsymbol{x}}{)}_{j}+{\mathop{\sigma }\limits^{ \sim }}^{2}))\\ {\mathscr{R}}({\boldsymbol{x}})\,:\,={\sum }_{j\in {\rm{\Omega }}}{\parallel {\mathscr{H}}}_{j}{\boldsymbol{x}}{\parallel }_{F}\end{array}$$

where ${\mathscr{C}}$ and Ω are sets of voxel indices, $\nu \in {{\mathbb{R}}}_{+}$ is a regularization parameter, $\parallel \,\cdot \,{\parallel }_{F}$ represents the Frobenius norm, and ${ {\mathcal H} }_{j}$ is the Hessian operator defined in Supplementary Section D. Note that it is possible to apply different weightings on the Hessian elements if the sampling rates differ in the the lateral and axial directions. However, we have used the standard Frobenius norm in our algorithm. A full derivation of this optimization problem is available in Supplementary Section C.

The non-negativity constraint is enforced using the indicator function ${ {\mathcal I} }_{\mathrm{[0,}+\infty )}\,:{{\mathbb{R}}}^{N}\to {\mathbb{R}}$, which is zero if all elements of the input are non-negative and infinity otherwise. For computational efficiency, the shift-invariant convolution expressed by the matrix ***A*** is implemented with the 3D fast Fourier transform (FFT). However, calculating a discrete convolution in the discrete Fourier domain makes implicit periodic signal assumptions that tend to produce large errors in the image formation model. These manifest as ringing artifacts in reconstructed volumes. Rather than relying on apodization or padding of captured images to suppress the error^7^, as many other algorithms do, we instead estimate a larger volume ${\boldsymbol{x}}\in {{\mathbb{R}}}^{N}$ than the observed focal stack ${\boldsymbol{y}}\in {{\mathbb{R}}}^{M}$; i.e., we pick *N* > *M*^19^. These extra pixels are allocated at the edges of the reconstructed volume, but we explicitly exclude the expanded area when evaluating the data fidelity term (i.e., we include only voxels in a region ${\mathscr{C}}$ corresponding to the original measured focal stack). The expanded edges in the reconstructed volume can then take whatever value is necessary to ensure that the voxels in ${\mathscr{C}}$ satisfy the data fidelity term as closely as possible. This approach reduces the mismodeling of the image formation model in the data fidelity while still retaining the computational efficiency of the FFT.

Both $\ell$ and ${\mathcal R}$ are convex functions so that the proposed deconvolution problem formulation (5) is a convex problem. While it can be solved by various algorithms, we use the alternating direction method of multipliers (ADMM)^10^. The computational complexity of this algorithm per iteration is ${\mathscr{O}}(N\,{\rm{l}}{\rm{o}}{\rm{g}}\,N)$, and the memory requirement is ${\mathscr{O}}(N)$. The complete iterative algorithm to solve problem (5) is given in Supplementary Section E. The iterations are stopped after the ${\ell }_{2}$-norm of the relative change is less than 10^−3^, or when the iteration number reaches 150.

### Performance comparison of noise models and regularization methods for simulated images

We turn our attention to assessing the performance of our deconvolution algorithm using simulated data. We added simulated noise to pristine images and fed these into our deconvolution algorithm. We compared results obtained using our shifted Poisson noise model and Hessian regularizer to deconvolution results for several other noise models and regularization strategies commonly used in deconvolution software. The problem splitting framework used in our optimization algorithm facilitated this comparison. By substituting in different data fidelity terms and regularizers (see Supplementary Section C), we were able to perform a direct comparison of these different methods within the same optimization framework.

Results for this quantitative comparisons are shown in Table 1 and Supplementary Table S3. Additional data related to these tests appear in Supplementary Figures S2–S8. The detailed simulation conditions are described in the Methods section. The definitions of each data fidelity and regularization term are given in Supplementary Tables S1 and S2.

**Table 1:** SNR comparison of different noise models and regularization methods.

Object	Number of maximum photoelectrons	Noise model (no regularizer)			3D Regularizer				
		Gaussian	Poisson	S-Poisson	${\ell }_{2}$	Laplacian	${\ell }_{1}$	TV	FH
Mitochondria	1000	−1.6	9.5	9.5	9.7	11.3	11.2	12.9	**13.6**
	500	−3.1	7.2	7.2	9.0	11.1	10.0	11.7	**12.5**
	100	−5.6	1.7	1.9	7.2	9.5	7.5	9.1	**9.9**
	50	−6.4	−0.7	−0.4	6.2	8.4	6.4	8.0	**8.7**
	10	−8.6	−6.0	−5.3	3.4	5.1	3.3	4.8	**5.5**
Actin	1000	−2.0	4.9	4.9	5.0	5.9	5.2	6.5	**6.9**
	500	−3.3	3.3	3.3	4.0	5.6	4.4	5.7	**6.1**
	100	−5.7	−0.8	−0.7	2.9	4.5	2.9	4.1	**4.6**
	50	−6.5	−2.6	−2.5	2.4	3.9	2.4	3.6	**4.0**
	10	−8.9	−6.9	−6.6	1.3	2.2	1.2	2.2	**2.7**
Nucleus	1000	−1.1	12.5	12.5	12.5	12.5	12.5	14.0	**15.0**
	500	−2.6	11.0	11.0	11.0	11.0	11.2	12.8	**13.8**
	100	−5.5	5.5	5.7	7.4	10.6	8.0	9.8	**10.7**
	50	−6.3	2.8	3.0	6.1	**9.2**	6.6	8.3	**9.2**
	10	−8.1	−3.7	−3.2	3.4	5.7	3.5	5.0	**5.8**

The performance of various noise models and regularizers are compared at different noise levels on simulated fluorescence images. The resulting SNRs are averaged over ten noise realizations and listed in the table in dB. The center columns of the table show a comparison of different noise models (Gaussian, Poisson, and shifted Poisson) where deconvolution is performed with the non-negativity constraint but without any regularizers. On the right side of the table, the shifted Poisson likelihood function is paired with different regularization methods. The ground truth images and the simulated images are shown in Supplementary Figure S2, and the deconvolved images are shown in Supplementary Figures S3–S8. The SNR is defined as $10\,{\mathrm{log}}_{10}(||{\boldsymbol{x}}|{|}_{2}^{2}/{\rm{MSE}})$, where ***x*** is the deconvolved image and MSE is the mean squared error between the original image and the deconvolved image.

First, we analyzed the effectiveness of different noise models. These comparisons, which appear on the left side of Table 1, were conducted across different noise levels without any spatial regularization. We found that the shifted Poisson noise model performed best at all noise levels among the three noise models we tested. This is to be expected since it best approximates the realistic Poisson-Gaussian noise model used to add noise to the images. The Gaussian noise model did not perform well at any noise level. The Poisson noise model performed well at high photon counts, but this analysis shows the shifted Poisson noise model to be more effective for low photon count images that are typically necessary in live cell imaging.

The right half of Table 1 shows signal-to-noise ratio (SNR) when the shifted Poisson noise model is used in conjunction with different regularization methods. We include the squared ${\ell }_{2}$ norm of a volume (i.e., Tikhonov regularization), the squared ${\ell }_{2}$ norm of a Laplacian filtered volume (Laplacian), the ${\ell }_{1}$ norm of a volume (i.e., Lasso regularization), the isotropic 3D total variation (TV) norm, and finally the Frobenius norm of the 3D Hessian (FH). See Supplementary Table S2 for a mathematical description of each method. All regularizers improved the image reconstruction quality as compared to non-regularized reconstruction. The ${\ell }_{2}$ norm performed worst amongst the compared regularizers. More effective were the ${\ell }_{1}$ and TV, which induce sparsity in the image domain and first derivative domain, respectively. The Laplacian and FH regularizer performed best at all noise levels. Both produced considerably improved reconstruction SNR even with very noisy input images, however the FH regularizer outperformed the Laplacian in almost all cases. This is because the Laplacian regularizer simply penalizes the high-frequency components while the FH regularizer promotes locally smooth structures. Eventually, the Laplacian smooths out all edges, but the FH tends to preserve edges.

We adopted SNR because it is widely used for comparing reconstructed images to ground truth and would thus provide the best basis for comparing our results to those of past deconvolution studies. However, we have also performed this analysis using a mutual information metric that may be better suited to comparing microscope images with Poisson noise statistics and heterogeneous noise variance from pixel to pixel. These results are in Supplementary Table S3. However, these results largely agree with the SNR analysis and both show that the FH regularizer combined with the shifted Poisson noise model is well suited to deconvolution problems involving biological structures and very low photon count imaging.

### Experimental validation

In this section, we demonstrate the performance of the proposed deconvolution method on experimentally-captured images. To show the effectiveness of our method, we have compared its results to two popular commercial software packages, Microvolution^8^ and Huygens, and two free software packages, ER-Decon 2^9^ and DeconvolutionLab2^7^. Specific algorithms and options we used in each software for this analysis are described in the Methods section. Pixel-value statistics of the raw images are provided in Supplementary Table S4.

#### Deconvolution of fluorescence microspheres

To evaluate the software on a simple known structure, a fluorescence microsphere was imaged with a widefield fluorescence microscope (see Methods for detailed imaging conditions). Since its three-dimensional structure is known, this type of microspheres is frequently used for the evaluation of deconvolution software^31^. Algorithmic parameters of each method were manually tuned by increasing the regularization until the noisy structures disappear in deconvolved images. This is the common practice in actual biological experiments, and we followed this strategy to provide a qualitative comparison.

**Figure 1:**
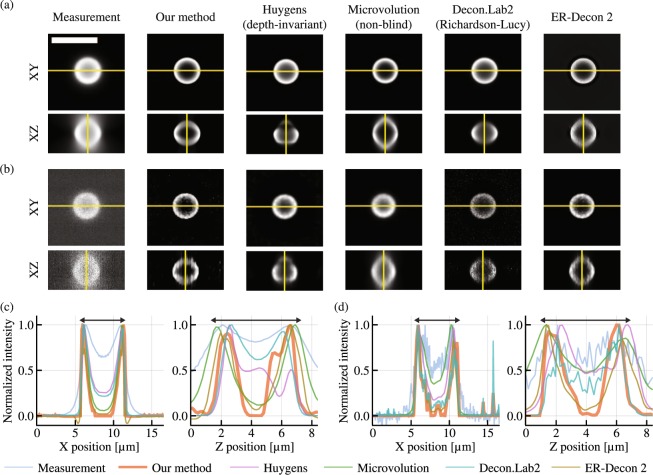
Deconvolution results for experimentally captured focal stacks containing a 6 μm hollow microsphere. (**a**) and (**b**) show the XY and XZ cross sections the 256 × 256 × 57 voxel focal stack (scale bar: 10 μm). Images with high and low photon counts are shown (see text for details), each deconvolved with multiple deconvolution algorithms. (**c**) and (**d**) show the intensity profile of the yellow lines in (**a**) and (**b**), respectively. Since each software has a different intensity scale, the intensity is normalized in (**c**) and (**d**) by dividing the intensity by its maximum value. The double-sided arrows in (**c**) and (**d**) have the length of 6 μm. All XY slices in the focal stacks are shown in a sequence in Supplementary Video 1.

With our method, the maximum values of the deconvolved images were 878.6 and 1.80 photoelectrons for the high and low photon-count images, respectively. These values confirm that the low photon-count image is extremely noisy. Figure 1 shows the results obtained from the evaluated deconvolution software, and whole volumes are visualized in Supplementary Video S1. The raw measurements have reduced contrast due to the out-of-focus light as well as axially elongated structure due to the shape of the widefield microscope’s PSF. After deconvolution, both lateral and axial cross sections are expected to have an empty core, a circular shape, and uniform intensity along the circumference.

Figure 1(a) and (b) show that all algorithms improved the contrast from the raw images. While DeconvolutionLab2 performed poorly on the low photon-count image, other software did not show significant degradation in image quality as compared to the high photon-count imaging case. We used the Richardson-Lucy algorithm for DeconvolutionLab2, and the performance degradation for noisy images was expected because this algorithm does not have a regularizer. On the other hand, the other software packages all use regularizers to make deconvolution more robust with noisy images. Notably, compared to other methods, the proposed method has enhanced contrast, suppressed axial elongation and uniform intensity along the circumferences. Although the axial cross section has a slightly distorted circular shape, the elongation is remarkably suppressed as shown in Fig. 1(c) and (d). The distortion may originate from the difference between the simulated PSF and the actual PSF of the microscope, but this could be improved with the measurement of the microscope’s PSF^32^.

#### Deconvolution of fixed fluorescence cells

**Figure 2:**
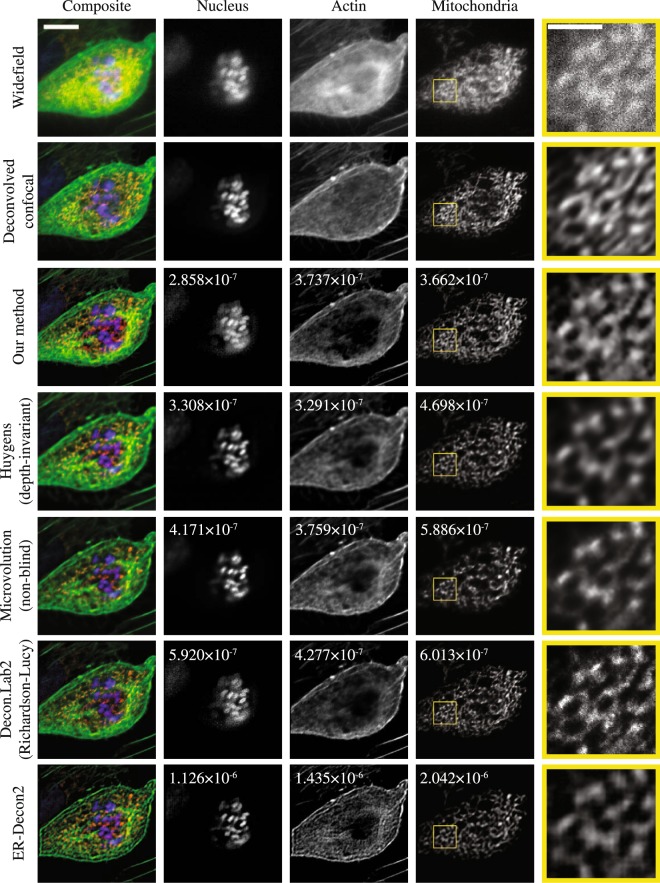
A comparison of 3D deconvolution software used to process a low SNR, 512 × 512 × 22 voxel widefield fluorescence focal stack. The focal stack was captured with three color channels (the first row) and then deconvolved with six deconvolution software methods (rows three through eight). The second row shows a confocal microscope image deconvolved with the Huygens software as a baseline for comparison. The fifth column shows the magnified image of the yellow rectangle in the corresponding mitochondria image. The composite images (the first column) show the sixth *z* slice of a pseudo-colored 3D image combining the three color channels. The nucleus, actin and mitochondria images (the second, third and fourth columns) show the eleventh, fourth and sixth *z* slice of the deconvolved 3D images. The scale bar in the composite image is 10 μm, and the scale bar in the magnified image is 3 μm. The number written in the figures are the minimum NMSE measured when comparing these data to deconvolved confocal images. All XY slices of the focal stacks are shown in a sequence in Supplementary Video 2.

Similarly, we evaluate the performance of our algorithm on a typical cellular sample whose mitochondria, nucleus, and actin filaments are stained with different fluorescence dyes. As each cellular component has distinct spatial structures, this sample is well suited for evaluating the versatility of different spatial regularizers. Unlike the fluorescent bead in the previous section, the ground truth structure is not known for this sample. Therefore, we captured the same cell with a spinning disk confocal microscope and deconvolved the confocal image with the Huygens software to obtain baseline images of the sample. Deconvolved confocal images were registered and aligned to the deconvolved widefield image with resampling and rotation based on bi-cubic interpolation. Figure 2 visualizes representative *z* slices of the measured images, the deconvolved confocal images, and the deconvolved widefield images. Whole volumes are visualized in Supplementary Video S2, and the detailed capture condition is summarized in the Methods section.

As mentioned in the previous section, each deconvolution software package (with the exception of DeconvolutionLab2) has its own unique regularization method, and each method exposes a user-tunable regularization parameter. To attempt a fair comparison of algorithm performance we tuned each regularization parameter to minimize the normalized mean squared error (NMSE) between the deconvolved images and the deconvolved confocal image. This way, each algorithm achieves the best possible fit to the same baseline data set prior to any comparisons between software packages.

**Figure 3:**
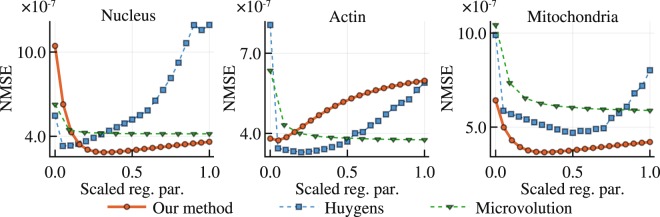
Tuning of regularization parameters. Normalized mean squared error (NMSE) values are plotted vs. regularization parameters. The NMSE measures the closeness of fit between the deconvolution result and a ground truth data set collected using a confocal microscope scan of the same fluorescent specimen. We evaluated how the choice of regularization parameter effects the NMSE for three deconvolution methods and three different samples with different morphologies. Because the magnitude of regularization parameters is different in different software, the parameters have been independently normalized to the range [0.0, 1.0]. We tested the following regularization parameters: 0.005, 0.010, 0.015, …, 0.100 for our method, 0, 1, 2, …, 20 for Huygens and 15, 105, 205, 305, 405, …, 1005 for Microvolution. Note that the regularization strength increases as the parameter increases in our software but decreases as the parameter increases in Huygens and Microvolution.

The NMSE is computed between the shown representative slices in Fig. 2, instead of the whole volumes. Normalization is performed by dividing the mean squared error by the ${\ell }_{2}$ norm of the deconvolved confocal image. Because each software handles intensity scaling differently, an offset adjustment and intensity scaling are applied to the deconvolved image before computing the NMSE. To find the optimal offset and scaling parameters that minimize the NMSE, we used an adaptive Nelder-Mead algorithm from the Optim.jl software package^33,34^. This NMSE evaluation is performed on images deconvolved with a series of regularization parameters and plotted for each software in Fig. 3 except ER-Decon 2, which has two regularization parameters rather than one. The reason why we used the representative slice, rather than the whole 3D volume, is that the accumulative NMSE over the volume is not representative of the reconstruction quality of individual slices. When using the whole volume for the minimization, the slices with less photons are found to be more weighted than the slices with the distinct structures so that the errors on the representative slices are not well minimized. For ER-Decon 2, we performed a 2D grid search to find the best pair based on the NMSE analysis (see Methods). However, the deconvolved images based on this search were visually not pleasant as shown in Supplementary Fig. S9 so that we have subjectively chosen the best images from the grid search and visualized them in Fig. 2.

After finding the optimal regularization parameter as described above, the minimized NMSE can be used as the similarity metric to evaluate the deconvolution performance. The minimized NMSE, listed in Fig. 2, shows that our software consistently outperformed other software in nucleus and mitochondria, but Huygens slightly outperformed in actin. However, for actin, all software misses fine filamentous structures. This may be partly due to the spatial regularization, but the mismatch between the actual PSF and the simulated PSF can also result in excess flattening. The images deconvolved with our method had a maximum value of 8.8, 84.0 and 9.7 photoelectrons in the nucleus, actin and mitochondria images, respectively.

#### Deconvolution of live fluorescence cells

**Figure 4:**
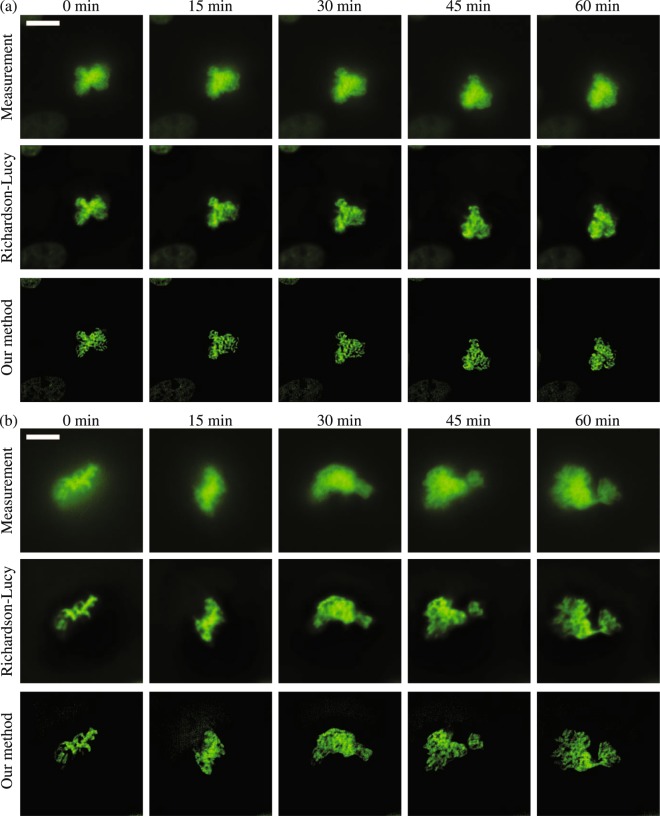
Deconvolution results for imaging of live cells expressing histone H2B fused to mClover to visualize chromosome conformation. (**a**) A focal stack (512 × 512 × 128 voxels) of the cell was captured over a span of one and a half hours with a time interval between focal stacks of three minutes. The 49-th *z* slice of the measurement, the deconvolved volume using our method, and the deconvolved volume using the Richardson-Lucy algorithm (100 iterations) is shown, with the time interval of fifteen minutes between frames along each row. (**b**) A second focal stack (512 × 512 × 100 voxels) shows a cell recorded for one hour with a time interval of three minutes. The 37-th *z* slice is shown, and the time interval between frames in each row is fifteen minutes. The corresponding deconvolved images are visualized in the second and third rows, respectively. The scale bar has the length of 10 μm. 3D volume renderings of these cells are provided as Supplementary Videos 3 and 4.

In this section we present a demonstration of our deconvolution algorithm in a live cell imaging application. As a baseline for comparison, our own implementation of the Richardson-Lucy algorithm was used to deconvolve the same dataset. For this experiment, cells expressing histone H2B fused to mClover are imaged to visualize the dynamics of chromosome conformation. Since the time-lapse images are captured with the same microscope, noise parameters were estimated for the first focal stack and then reused for all other focal stacks. Figure 4 shows a representative slice of the raw images and the deconvolved images. Our method resulted in substantially sharper deconvolved images than the Richardson-Lucy method. The deconvolved images at 0 min had the maximum value of 24.3 and 13.3 photoelectrons in Fig. 4(a) and (b), respectively. The volumes of the time-lapse images are rendered with Imaris (version 9.0) and provided as Supplementary Videos S3 and S4.

## Discussion

We have demonstrated that our deconvolution algorithm produces 3D reconstructions with low overall NMSE when imaging at extremely low light levels. The combination of the accurate Poisson-Gaussian noise model and the Hessian-based regularizer was shown to outperform other common noise models and regularization strategies. Our method also compares favorably to other free and commercially available deconvolution algorithms when tested on a variety of experimentally collected data.

Ours is not the first algorithm to address the need for deconvolution algorithms capable of processing low SNR datasets. Previously, Arigovindan *et al*. proposed an algorithm that uses a Hessian-based regularizer formulated using an entropy-based approach, a negativity penalty term and an ${\ell }_{2}$-norm data fidelity term, which assumes Gaussian noise^9^. The Gaussian noise assumption is commonly used for high photon-count conventional photographic images but is not applicable to low photon-count images. However, due to their Hessian-based regularizer, they reported its successful application to low photon-count images. On the other hand, their method was not convex and, therefore, does not guarantee its convergence to a globally optimal solution while our convex algorithm does. In addition, their algorithm uses two regularization parameters rather than one, which results in difficult tuning on real-world data sets.

Our algorithm could be improved in several ways. First, we assumed uniformity of noise characteristics over all pixels for formulating the data fidelity term. If we measure the noise characteristics on a pixel-by-pixel basis, we could easily extend the data fidelity term to incorporate pixel-dependent noise characteristics of CMOS cameras. Moreover, we could handle dead and hot pixels by excluding them from the data fidelity term without modifying other parts of the algorithm. Second, we have used a simulated depth-invariant PSF based on a theoretical model that does not account for optical aberrations. While our algorithm worked well on experimentally captured images, the slightly-distorted circular shape for the fluorescence bead (Fig. 1) suggests that this PSF model may be slightly inaccurate for our microscope. In practice, the microscope’s PSF includes some amount of optical aberration due the microscope optics or refractive index variations and scattering in the sample itself.

Another case requiring a depth-variant PSF is when there is any refractive index mismatch between the immersion medium and the observed sample. Assuming that the sample has a reasonably uniform refractive index, a depth-varying PSF can be simulated using the Gibson and Lanni model^35^. This technique has been successfully applied in other deconvolution studies^36–39^. Our deconvolution formulation could be similarly adapted to use this or other depth-varying PSFs by simply changing the image formation model. Alternatively, an empirical model based on real measurements of the PSF is sometimes used in place of a theoretical model to better capture such non-ideal imaging conditions^32^. It would be straight-forward to use an empirical model in our algorithm. We also note that blind deconvolution enables one to improve the PSF during deconvolution to better model aberrations that are not known *a priori*. Both of these approaches are implemented in some commercial software. Supplementary Figure S11 shows that these approaches have the potential for improving the performance of our method, and this could be an area for future work. Finally, we note that changes to our PSF model would also enable our deconvolution algorithm to be adapted to other imaging modalities such as confocal microscopy and light-sheet microscopy.

## Methods

### Simulation of fluorescence cells

In order to obtain 3D cellular structures for use in our simulated tests, we used fixed fluorescence cells (FluoCells Prepared Slide #1, Thermo Fisher Scientific). Samples were imaged with a Zeiss LSM 780 laser-scanning confocal microscope. The captured images were convolved with a 2D Gaussian filter, small intensity values were clipped to zero, and the images were subsampled to further suppress noise. The processed images were cropped to 256 × 256 × 12 and were zero-padded along the optical axis to the size of 22 slices. The resulting images were regarded as noise-free ground truth images for the purposes of simulating the image capture process. These ground truth cellular images were convolved in the frequency domain with a PSF simulated with a size of 256 × 256 × 22 with an N.A. of 1.4, a wavelength of 525 and a immersion medium’s refractive index of 1.51. The lateral and axial sampling rate in simulation were set to 65 nm/px and 150 nm/px, respectively. Image noise was simulated as the mixed Poisson-Gaussian noise with *γ* = 2.0 and *σ* = 3.0 which represents realistic noise statistics of a CCD camera. To maximize the SNR with a given regularizer, the regularization weight *ν* is searched with the golden section search until the search gap becomes less than 10^−5^. On high-photon-count images, ${\ell }_{2}$-norm and Laplacian regularizers were not helpful for deconvolution, and this search resulted in a tuning that effectively disabled regularization altogether.

### Capture condition of a fluorescence microsphere

A fluorescence microsphere with a diameter of 6 µm (FocalCheck Fluorescence Microscope Test Slide #1, Thermo Fisher Scientific) illuminated with a mercury lamp was imaged with an PLAN APO 100X 1.4 N.A. oil-immersion objective lens with a motorized XYZ-axis stage (MS-2000, Applied Scientific Instrumentation). The microscope was operated with the *μ* Manager software^40^. Images were recorded with a scientific CMOS camera (ORCA-Flash 4.0 V2, Hamamatsu Photonics). The core and shell of the microsphere were stained with blue and green fluorescence, respectively, but captured only through a green fluorescence 540/40 nm filter cube. The microsphere was embedded under a coverslip in a medium with a refractive index of approximately 1.52. The lateral and axial sampling rate were set to 65 nm/px and 150 nm/px, respectively.

### Capture condition of fixed fluorescence cells

For the fixed fluorescence cells, FluoCells Prepared Slide #1 (Thermo Fisher Scientific) were used. This slide is prepared with bovine pulmonary artery endothelial cells and is stained with DAPI, Alexa Fluor 488 phalloidin and MitoTracker Red CMXRos for staining nuclei, actin and mitochondria, respectively. Images were captured using a Nikon Eclipse-TI inverted microscope with an oil immersion PLAN APO-TIRF 100X 1.49 N.A. objective lens and a piezo Z-axis stage (Mad City Labs). For the immersion oil, Immersol 518 F (Carl Zeiss) with refractive index 1.518 was used. This microscope is equipped with a conventional widefield microscopy system and the Yokogawa CSU-X1 spinning disk confocal system on different light paths. The widefield microscopy system is equipped with an Andor Neo sCMOS camera (Andor Technology) and a 300 W xenon arc light source (Lambda XL, Sutter Instrument). With this system, fluorophores can be excited with a 387/11 nm, 490/20 nm and 555/25 nm excitation filter and collected with a 455/50 nm, 525/36 nm and 605/52 nm emission filter, respectively. The lateral and axial sampling rate were set to 65 nm/px and 150 nm/px, respectively, and the camera is operated with 11-bit depth. The spinning disk confocal microscope system is equipped with an Andor iXon Ultra-897 EM-CCD camera (Andor Technology). With this confocal system, fluorophores can be excited with a 405 nm, 488 nm, 561 nm laser and collected with 445/20 nm, 525/30 nm and 600/37 nm emission filters, respectively.

### Capture condition of live fluorescence cells

HeLa S3 cells expressing histone H2B fused to mClover under CMV promoter were maintained in DMEM supplemented with 10% FBS (Omega Scientific), 2 mM Glutamine (Life Technologies) and 1 × Penicillin/Streptomycin (Life Technologies) at 37 °C 5% CO_2_. Cells were plated in Fluorodish (World Precision Instruments) at 0.5 × 10^6^ cells/well 24 h prior to imaging. The media was replaced to Fluorobrite (Thermo Fisher Scientific) with 10 mM Hepes, 1% FBS and 2 mM Glutamine 1 h prior to imaging. The imaging was performed by the same microscope with the fixed cell experiment in a 37 °C 5% CO_2_ chamber. In this experiment, the widefield images were captured with a PLAN APO IR 60X 1.27 N.A. water immersion lens and a 1.5X zoom lens with 20 ms exposure time per frame. For the immersion medium, Immersol W (Carl Zeiss) with refractive index 1.334 was used. The lateral and axial sampling rate were set to 72 nm/px and 200 nm/px, respectively, and the images were captured at a time interval of 3 min.

### Pre-processing of captured images

Before applying our deconvolution method, captured images are pre-processed with dark-field correction and flat-field correction procedures^30^. After subtracting an offset image or a dark-field image, images were divided by a flat-field image in a pixel-by-pixel basis. The dark-field image was prepared by computing the sample mean from 1000 dark frames. The flat-field image was acquired for each combination of an objective lens and a filter set using the following procedure. First, an autofluorescence plastic slide is imaged at 100 different positions, and their median values are computed at each pixel. Subsequently, the median image is subtracted by the dark image. To compensate for the exposure time the resulting image is divided by the median value of the entire image to produce a flat-field image.

### Usage of deconvolution software

We used Huygens (version 17.10, Scientific Volume Imaging), Microvolution (version 2017.06, Microvolution), ER-Decon 2 in Priism (version 4.6.1, UCSF Macromolecular Structure Group) and DeconvolutionLab2 (version 08.05.2017, EPFL Biomedical Imaging Group). For ER-Decon 2 and DeconvolutionLab2, we used the same PSF model as was used in our own deconvolution algorithm. Because Huygens and Microvolution are equipped with their own PSF model, we used their simulated PSF for deconvolution. Although Huygens has a depth-varying PSF model and Microvolution has a blind deconvolution mode, we have used a depth-invariant PSF model for Huygens and non-blind deconvolution mode for Microvolution for our series of comparisons because our own algorithm uses a depth-invariant PSF model and non-blind deconvolution. Deconvolution results with Huygens depth-varying PSF and Microvolution blind deconvolution are provided in Supplementary Figure S11.

Among the collection of classical deconvolution algorithms in DeconvolutionLab2, we used the Richardson-Lucy deconvolution algorithm with 10-pixel zero-padding after scaling the captured image to be in calibrated units of photoelectrons. For Microvolution, ER-Decon 2 and DeconvolutionLab2, the iterations are fixed to 100 as they do not have convergence-check functionality. For Huygens, we used the Classic Maximum Likelihood Estimation with one brick and fully-padded options. The software was run until the image quality criterion reaches 0.01. It typically converged within 100 iterations.

For Microvolution, we used 10-pixel padding for each direction and the maximum entropy regularization with the non-blind mode. Note that while our method increases the strength of the regularization as the regularization parameter increases, Huygens and Microvolution increases their regularization as the parameter *decreases*. For ER-Decon 2, we used 10-pixel padding and all other parameters set to default values. For this reason, we obtained some negative values in the deconvolved images. Although the negative values could be removed by tuning the positivity regularization parameter, it may not improve image quality. For the 2D grid search, the smoothing parameter was chosen from 24 parameters, 1.0, 2.0, 5.0 × 10^−5,−4,−3,−2,−1,0,1,2^, and the nonlinear parameters was chosen from 14 parameters, $1.0\times {10}^{\mathrm{0,1,2,}\ldots \mathrm{,13}}$. Every single pair of the regularization parameters was used for the deconvolution.

Our software, Huygens, ER-Decon 2 and DeconvolutionLab2 were run on a machine with 3.6 GHz Intel Core i7-4790 CPU. Additionally, Huygens and our software are accelerated with an NVIDIA Quadro K6000. Due to the availability of the software license, Microvolution was run on a different machine with a 3.4 GHz Intel Xeon E5-2687 v2 and an NVIDIA Quadro K5000.

The runtimes for each software deconvolving the focal stack of the mitochondria in Fig. 2 were 84.2 s for our software (140 iterations), 2.4 s for Huygens (32 iterations), 4.7 s for Microvolution (100 iterations), 121.1 s for ER-Decon 2 (100 iterations) and 284.8 s for DeconvolutionLab2 (100 iterations). Note that the runtimes of Huygens and Microvolution include the time to simulate a PSF.

### Visualization of images

All images are saved as single-precision floating-point images. For the visualization of the cross-sectional views, each image is exported as a 8-bit image after scaling the image so that 0.01 of pixels are saturated at 0 and 255.

### Data availability

The dataset used in this study and the source code for our software is available at https://github.com/computational-imaging/3Deconv.jl

## Electronic supplementary material

Supplementary Video S1

Supplementary Video S2

Supplementary Video S3

Supplementary Video S4

Supplementary material
