# Supplementary material for: A convex 3D deconvolution algorithm for low photon count fluorescence imaging
Source: Sci Rep. 2018 Jul 31;8:11489. doi: 10.1038/s41598-018-29768-x (PMC6068180; doi:10.1038/s41598-018-29768-x)
Supplement: Supplementary file 5 — Supplementary material [file 41598_2018_29768_MOESM5_ESM.pdf]

A convex 3D deconvolution algorithm for low photon count fluorescence imaging — Supplementary material

Hayato Ikoma[1], Michael Broxton[1], Takamasa Kudo[2], and Gordon Wetzstein[1,*]

[1]Stanford University, Department of Electrical Engineering, Stanford, 94305, United States
[2]Stanford University, Department of Chemical and Systems Biology, Stanford, 94305, United States
[*]Correspondence to gordon.wetzstein@stanford.edu

A Definition of the transform $\mathcal{F}$

The transform $\mathcal{F}$ mathematically models the optical Fourier transform that is performed by a lens. It is defined as

$$\mathcal{F}\{U(u,v)\}(x,y) := \int_{-\infty}^{\infty}\int_{-\infty}^{\infty} U(u,v)\exp\left\{-i\frac{2\pi}{\lambda f_{\mathrm{tl}}}(ux+vy)\right\} du\, dv, \tag{S1}$$

where $U(u,v)$ denotes the input wave field at $(u,v) \in \mathbb{R}^2$, f_{tl} is the focal length of the tube lens performing the optical Fourier transform, and λ is the wavelength of a light[1]. This transform can be considered as the conventional Fourier transform with a scaling of the coordinates.

B Derivation of the data fidelity $\ell(\boldsymbol{x})$

Given the latent fluorescence volume $\boldsymbol{x} \in \mathbb{R}^N$ and the read noise standard deviation $\tilde{\sigma}$, the joint probability density function of the N-dimensional random vector $\boldsymbol{Y}$ based on the shifted Poisson noise model (4) is

$$f_{\boldsymbol{Y}}(\boldsymbol{y}) = \prod_{j\in\mathcal{C}} \frac{((\boldsymbol{Ax})_j + \tilde{\sigma}^2)^{\boldsymbol{y}_j+\tilde{\sigma}^2} e^{-((\boldsymbol{Ax})_j+\tilde{\sigma}^2)}}{(\boldsymbol{y}_j + \tilde{\sigma}^2)!}, \tag{S2}$$

where the set $\mathcal{C}$ represents the indices of the focal stack. Then, we define the data fidelity for $\boldsymbol{x}$ in our deconvolution problem as the negative natural logarithm of the probability distribution given the measurements $\tilde{\boldsymbol{y}}$:

$$\ell(\boldsymbol{x}) := \sum_{j\in\mathcal{C}} \left((\boldsymbol{Ax})_j - (\tilde{\boldsymbol{y}}_j + \tilde{\sigma}^2)\log((\boldsymbol{Ax})_j + \tilde{\sigma}^2)\right). \tag{S3}$$

Here, we dropped the constant term which does not affect the solution of the optimization problem. The convexity of $\ell(\boldsymbol{x})$ is guaranteed if $\tilde{\boldsymbol{y}}_j + \tilde{\sigma}^2 \geq 0$ is satisfied for all $j \in \mathcal{C}$. Note that when the measurements do not satisfy these convexity condition, we clip these measurements $\tilde{\boldsymbol{y}}_j$ to $-\tilde{\sigma}^2$ to enforce the convexity condition. This clipping procedure is known in the field of X-Ray computed tomography to produce bias to the reconstruction if the clipping is performed on large number of voxels[2]. However, scientific CMOS cameras used in fluorescence microscopy have extremely low readout noise, so we do not expect bias to be a major problem in our algorithm. We found that only 1% of reconstructed voxels are clipped to zero in a typical volume in simulation.

C Definitions of data fidelity terms for different noise models, and different regularizers

Supplementary Tables S1 and S2 show the different data fidelity terms and regularization terms used to produce Table 1 and Supplementary Table S3 in the main manuscript.

D Definition of the linear operators

The linear operator $\mathcal{D}_j$ in Supplementary Table S2 is defined as

$$\mathcal{D}_j\boldsymbol{\theta} := \left[(\boldsymbol{D}_x\boldsymbol{\theta})_j \quad (\boldsymbol{D}_y\boldsymbol{\theta})_j \quad (\boldsymbol{D}_z\boldsymbol{\theta})_j\right]^T, \tag{S4}$$

Data fidelity	$\ell(\boldsymbol{x})$
Gaussian noise model	$\sum_{j\in\mathcal{C}} ((\boldsymbol{Ax})_j - \tilde{\boldsymbol{y}}_j)^2$
Poisson noise model	$\sum_{j\in\mathcal{C}} ((\boldsymbol{Ax})_j - \tilde{\boldsymbol{y}}_j \log(\boldsymbol{Ax})_j)$
Shifted Poisson noise model	$\sum_{j\in\mathcal{C}} \big((\boldsymbol{Ax})_j - (\tilde{\boldsymbol{y}}_j + \tilde{\sigma}^2)\log((\boldsymbol{Ax})_j + \tilde{\sigma}^2)\big)$

Supplementary Table S1. List of the data fidelity terms $\ell(\boldsymbol{x})$ based on different noise models used in Table 1. Each data fidelity term can be derived similarly to equation (S3) based on the probability distribution of each noise model.

Regularizer	$\mathcal{R}(\boldsymbol{x})$
Squared ℓ_2 norm	$\sum_{j\in\Omega} \boldsymbol{x}_j^2$
Laplacian	$\sum_{j\in\Omega} (\boldsymbol{Lx})_j^2$
ℓ_1 norm	$\sum_{j\in\Omega} \lvert\boldsymbol{x}_j\rvert$
Isotropic TV norm	$\sum_{j\in\Omega} \lVert\mathcal{D}_j\boldsymbol{x}\rVert_2$
Frobenius norm of Hessian	$\sum_{j\in\Omega} \lVert\mathcal{H}_j\boldsymbol{x}\rVert_F$

Supplementary Table S2. List of the regularization terms $\mathcal{R}(\boldsymbol{x})$ used in Table 1. The definitions of the linear operators $\boldsymbol{L}$, $\mathcal{D}_j$ and $\mathcal{H}_j$ are given in equations (S4) and (S5).

where $\boldsymbol{D}_x$, $\boldsymbol{D}_y$, $\boldsymbol{D}_z$ are linear operators that compute the forward finite difference approximations of the first-order derivative of a 3D image along each direction. The Hessian linear operator $\mathcal{H}_j$ can then be defined in terms of these finite difference operators.

$$\mathcal{H}_j\boldsymbol{\theta} := \begin{bmatrix} (\boldsymbol{D}_{xx}\boldsymbol{\theta})_j & (\boldsymbol{D}_{xy}\boldsymbol{\theta})_j & (\boldsymbol{D}_{xz}\boldsymbol{\theta})_j \\ (\boldsymbol{D}_{yx}\boldsymbol{\theta})_j & (\boldsymbol{D}_{yy}\boldsymbol{\theta})_j & (\boldsymbol{D}_{yz}\boldsymbol{\theta})_j \\ (\boldsymbol{D}_{zx}\boldsymbol{\theta})_j & (\boldsymbol{D}_{zy}\boldsymbol{\theta})_j & (\boldsymbol{D}_{zz}\boldsymbol{\theta})_j \end{bmatrix}, \tag{S5}$$

where $\boldsymbol{D}_{ij} = \boldsymbol{D}_i\,\boldsymbol{D}_j$ for $i, j \in (x, y, z)$ are linear operators that compute the forward finite difference approximations of the second-order derivative of a 3D image along each direction. The Laplacian $\boldsymbol{L}$ is defined as

$$\boldsymbol{L\theta} := \boldsymbol{D}_{xx}\boldsymbol{\theta} + \boldsymbol{D}_{yy}\boldsymbol{\theta} + \boldsymbol{D}_{zz}\boldsymbol{\theta}. \tag{S6}$$

Note that while these finite difference operators are normally most efficient to compute in the primal domain, we apply them in the Fourier transform domain because they can be combined with transform domain operations necessary to solve one of the ADMM subproblems, as described below.

E ADMM solver for the proposed deconvolution problem formulation

The proposed problem formulation for deconvolution can be solved by various convex algorithms. In this study, we use the alternating direction method of multipliers (ADMM) to solve the problem[3]. To derive the algorithm, we first define a linear operator $\boldsymbol{H} \in \mathbb{R}^{6N\times N}$ where

$$[\boldsymbol{H\theta}]_j^T := \big[(\boldsymbol{D}_{xx}\boldsymbol{\theta})_j \quad (\boldsymbol{D}_{yy}\boldsymbol{\theta})_j \quad (\boldsymbol{D}_{zz}\boldsymbol{\theta})_j \quad (\sqrt{2}\boldsymbol{D}_{xy}\boldsymbol{\theta})_j \quad (\sqrt{2}\boldsymbol{D}_{xz}\boldsymbol{\theta})_j \quad (\sqrt{2}\boldsymbol{D}_{yz}\boldsymbol{\theta})_j\big]^T. \tag{S7}$$

This redefined linear operator is for notational convenience. It has the property that $\lVert\mathcal{H}_j\boldsymbol{\theta}\rVert_F = \lVert[\boldsymbol{H\theta}]_j\rVert_2$, because $\boldsymbol{D}_{xy}\boldsymbol{\theta} = \boldsymbol{D}_{yx}\boldsymbol{\theta}$, $\boldsymbol{D}_{xz}\boldsymbol{\theta} = \boldsymbol{D}_{zx}\boldsymbol{\theta}$, $\boldsymbol{D}_{yz}\boldsymbol{\theta} = \boldsymbol{D}_{zy}\boldsymbol{\theta}$. This allows us to rewrite the proposed problem more concisely by introducing auxiliary variables $\boldsymbol{z}_1 \in \mathbb{R}^N$, $\boldsymbol{z}_2 \in \mathbb{R}^N$ and $\boldsymbol{z}_3 \in \mathbb{R}^{6N}$:

$$\text{minimize} \quad \sum_{j\in\mathcal{C}} \big(\boldsymbol{z}_{1j} - (\tilde{\boldsymbol{y}}_j + \tilde{\sigma}^2)\log(\boldsymbol{z}_{1j} + \tilde{\sigma}^2)\big) + \mathcal{I}_{[0,+\infty)}(\boldsymbol{z}_2) + \nu\sum_{j\in\Omega} \lVert[\boldsymbol{z}_3]_j\rVert_2 \quad \text{such that} \begin{cases} \boldsymbol{Ax} = \boldsymbol{z}_1 \\ \boldsymbol{x} = \boldsymbol{z}_2 \\ \boldsymbol{Hx} = \boldsymbol{z}_3 \end{cases}. \tag{S8}$$

This convex, equality-constrained problem can now be solved with ADMM. On each ADMM iteration, we compute the following update steps:

$$\boldsymbol{x}^{k+1} := \underset{\boldsymbol{x}}{\operatorname{argmin}} \frac{\rho}{2} \left(\|\boldsymbol{A}\boldsymbol{x} - \boldsymbol{z}_1^k + \boldsymbol{u}_1^k\|_2^2 + \|\boldsymbol{x} - \boldsymbol{z}_2^k + \boldsymbol{u}_2^k\|_2^2 + \|\boldsymbol{H}\boldsymbol{x} - \boldsymbol{z}_3^k + \boldsymbol{u}_3^k\|_2^2 \right) \tag{S9}$$

$$\boldsymbol{z}_1^{k+1} := \underset{\boldsymbol{z}_1}{\operatorname{argmin}} \left(\sum_{j \in \mathcal{C}} \left({\boldsymbol{z}_1}_j - (\tilde{\boldsymbol{y}}_j + \tilde{\sigma}^2) \log({\boldsymbol{z}_1}_j + \tilde{\sigma}^2) \right) + \frac{\rho}{2} \|\boldsymbol{A}\boldsymbol{x}^{k+1} - \boldsymbol{z}_1 + \boldsymbol{u}_1^k\|_2^2 \right) \tag{S10}$$

$$\boldsymbol{z}_2^{k+1} := \underset{\boldsymbol{z}_2}{\operatorname{argmin}} \left(\mathcal{I}_{[0,+\infty)}(\boldsymbol{z}_2) + \frac{\rho}{2} \|\boldsymbol{x}^{k+1} - \boldsymbol{z}_2 + \boldsymbol{u}_2^k\|_2^2 \right) \tag{S11}$$

$$\boldsymbol{z}_3^{k+1} := \underset{\boldsymbol{z}_3}{\operatorname{argmin}} \left(\nu \sum_{j \in \Omega} \|[\boldsymbol{z}_3]_j\|_2 + \frac{\rho}{2} \|\boldsymbol{H}\boldsymbol{x}^{k+1} - \boldsymbol{z}_3 + \boldsymbol{u}_3^k\|_2^2 \right) \tag{S12}$$

$$\boldsymbol{u}_1^{k+1} := \boldsymbol{u}_1^k + \boldsymbol{A}\boldsymbol{x}^{k+1} - \boldsymbol{z}_1^{k+1} \tag{S13}$$

$$\boldsymbol{u}_2^{k+1} := \boldsymbol{u}_2^k + \boldsymbol{x}^{k+1} - \boldsymbol{z}_2^{k+1} \tag{S14}$$

$$\boldsymbol{u}_3^{k+1} := \boldsymbol{u}_3^k + \boldsymbol{H}\boldsymbol{x}^{k+1} - \boldsymbol{z}_3^{k+1}, \tag{S15}$$

where $\rho > 0$ is the ADMM parameter, which we will discuss later in this section. The superscript k represents the iteration number, and $\boldsymbol{u}_1 \in \mathbb{R}^N$, $\boldsymbol{u}_2 \in \mathbb{R}^N$ and $\boldsymbol{u}_3 \in \mathbb{R}^{6N}$ are the scaled dual variables[3]. These subproblems for updating $\boldsymbol{x}$, $\boldsymbol{z}_1$, $\boldsymbol{z}_2$ and $\boldsymbol{z}_3$ have closed solutions. As the subproblem (S9) is a linear least-square problem, the solution is

$$\boldsymbol{x}^{k+1} = (\boldsymbol{A}^T\boldsymbol{A} + \boldsymbol{I}^T\boldsymbol{I} + \boldsymbol{H}^T\boldsymbol{H})^{-1} \left(\boldsymbol{A}^T(\boldsymbol{z}_1^k - \boldsymbol{u}_1^k) + \boldsymbol{I}^T(\boldsymbol{z}_2^k - \boldsymbol{u}_2^k) + \boldsymbol{H}^T(\boldsymbol{z}_3^k - \boldsymbol{u}_3^k) \right). \tag{S16}$$

The operators $\boldsymbol{I}$, $\boldsymbol{A}$ and $\boldsymbol{H}$ are circulant matrices, so this closed-form formula can be efficiently computed through the diagonalization by the 3D discrete Fourier transform. The subproblem (S10) can be solved by finding a value $\boldsymbol{z}_1$ that causes its first derivative to go to zero:

$${\boldsymbol{z}_1}_j^{k+1} = \begin{cases} -\frac{1-\rho((\boldsymbol{A}\boldsymbol{x}^{k+1}+\boldsymbol{u}_1^k)_j - \tilde{\sigma}^2)}{2\rho} + \sqrt{\left(\frac{1-\rho((\boldsymbol{A}\boldsymbol{x}^{k+1}+\boldsymbol{u}_1^k)_j - \tilde{\sigma}^2)}{2\rho}\right)^2 + \frac{\tilde{\boldsymbol{y}}_j + \rho\tilde{\sigma}^2(\boldsymbol{A}\boldsymbol{x}^{k+1}+\boldsymbol{u}_1^k)_j}{\rho}} & \text{for } j \in \mathcal{C} \\ (\boldsymbol{A}\boldsymbol{x}^{k+1} + \boldsymbol{u}_1^k)_j & \text{for } j \notin \mathcal{C} \end{cases}. \tag{S17}$$

The subproblem (S11) is simply the projection onto the non-negative orthant:

$${\boldsymbol{z}_2}_j^{k+1} = \begin{cases} \boldsymbol{x}_j^{k+1} + {\boldsymbol{u}_2}_j^k & \text{if } \boldsymbol{x}_j^{k+1} + {\boldsymbol{u}_2}_j^k > 0 \\ 0 & \text{otherwise} \end{cases}. \tag{S18}$$

Finally, the subproblem (S12) can be computed using block soft-thresholding[4,5]:

$$[\boldsymbol{z}_3^{k+1}]_j = \max\left\{ \left(1 - \frac{\nu/\rho}{\|[\boldsymbol{H}\boldsymbol{x}^{k+1} + \boldsymbol{u}_3^k]_j\|_2} \right) [\boldsymbol{H}\boldsymbol{x}^{k+1} + \boldsymbol{u}_3^k]_j,\, 0 \right\} \tag{S19}$$

In our software, $\boldsymbol{x}, \boldsymbol{z}_1, \boldsymbol{z}_2, \boldsymbol{z}_3, \boldsymbol{u}_1, \boldsymbol{u}_2, \boldsymbol{u}_3$ are initialized to be zero. The ADMM parameter ρ is set to $\rho = 6000\nu/\max(\tilde{\boldsymbol{y}})$. Our choice for this parameter is similar to the method described by Lefkimmiatis et. al[6]. However, their proposed value for ρ worked well on simulated images but not for experimentally-captured images. Once chosen, our value for ρ performed well for both our simulated and experimental data sets.

For the use of other data fidelity functions and regularizers shown in Supplementary Tables S1 and S2, we simply modified the above update rules to solve each problem formulation using the data fidelity and regularization functions from those tables. In cases where we don't enforce any regularization, the ADMM parameter is set to $\rho = 6/\max(\tilde{\boldsymbol{y}})$. For more information about the flexibility of linear image restoration algorithms based on proximal algorithms (including ADMM) we refer the interested reader to these papers by Heide et al.[7,8]. This algorithm is implemented with Julia v0.6[9] and is accelerated with a GPU by using the ArrayFire library[10].

F Performance validation of the noise estimation

With the Poisson-Gaussian noise model (2), the camera noise statistics can be modeled with two parameters: the camera gain γ and the read noise standard deviation σ. In this section we compare the noise parameters γ and σ estimated with the classical

method, in which these parameters are measured from calibration images, to those estimated using our automated approach that is based on Foi's method[11] which estimates these parameters directly from the focal stacks that we reconstruct with our deconvolution algorithm. Foi's method simplifies data collection for deconvolution, because it does not require collecting extra data prior to an experiment.

In the classical method, a dark frame image is captured without any incoming light and read noise is estimated by computing the standard deviation σ of a 200×200 image patch. To estimate the camera gain γ, autofluorescent plastic slides are imaged at thirteen different exposure times and the sample variances for each exposure level is computed over a 200×200-pixel image patch. Then, a linear fit is computed for these exposure time/sample variance data points. The slope of this fit is used as the camera gain γ. The classical fit we computed for our scientific camera is the orange line in Supplementary Figure S1.

We implemented our own version of Foi's noise estimation algorithm to estimate the noise statistics[11]. This algorithm estimates γ and σ from a single-shot conventional photograph or a single focal stack. Although this method was originally designed for photographic camera images, we found it to work well with fluorescence microscope focal stacks so long as they contain enough structure to permit accurate parameter estimation. We note that since the flat-field correction effectively changes the camera gain and compensates the pixel variation of the gain, all of these estimations are performed after the flat-field correction of these images.

To test the stability of Foi's algorithm, we ran it on 164 different focal stacks $512 \times 512 \times 15$ of a fluorescence cellular sample. Each focal stack was captured at different positions in the sample slide so that each contained different image content. Two example inputs are shown in Supplementary Figure S1(a). In Supplementary Figure S1(b) we show a histogram of estimates of γ and σ computed using Foi's algorithm for these 164 trials. These histograms can then be compared to an estimate we obtained for our camera using the classical method. Estimates using Foi's algorithm closely matched the classical method. Specifically, using the classical method we obtained the estimated noise parameters of $\gamma = 2.30$ and $\sigma = 2.37$. The average and standard deviation of all 164 trials using Foi's method was $\gamma = 2.39 \pm 0.23$ and $\sigma = 2.52 \pm 0.37$.

We did note one problem with Foi's algorithm in our tests. It does not enforce the non-negativity of the standard deviation σ, and occasionally a negative σ can be obtained. We believe this can occur due to hot pixels and dead pixels on the camera sensor that violate the noise assumptions made by Foi's algorithm. In such situations, we note that σ can still be estimated from a single dark frame using the traditional approach, and then γ can still be estimated from a focal stack using Foi's algorithm.

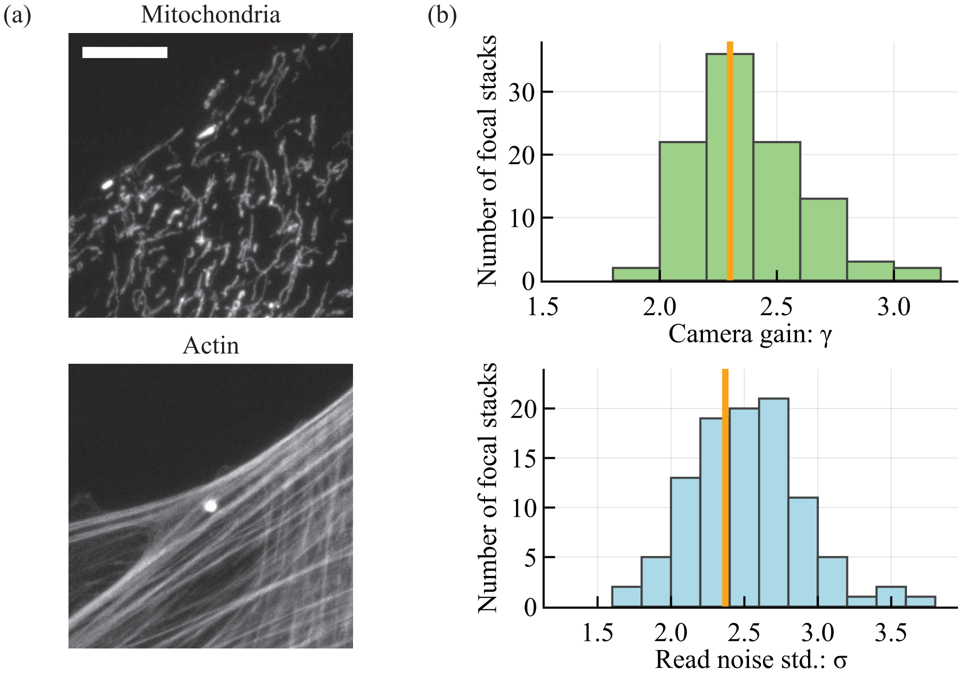

Supplementary Figure S1. Performance validation of Foi's noise estimation algorithm. (a) Representative slices from two experimentally-captured $512 \times 512 \times 15$-pixel focal stacks. (scale bar: $10\,\mu m$) (b) 164 focal stacks were independently evaluated using Foi's method. These histograms show the estimated camera gain γ and read noise standard deviation σ computed for each focal stack. These are closely clustered around the parameters estimated using the classical, manual calibration approach (orange lines, $\gamma = 2.30$ and $\sigma = 2.37$).

G Performance comparison of different noise models and regularization methods

This section provides an alternative analysis of the results in Table 1 of the main manuscript. Here we have used mutual information, rather than SNR, to compare reconstructed images to our ground truth data set[12, 13]. We include this analysis because in low photon count images we expect that pixel-by-pixel errors do not follow the Gaussian distribution, and therefore the mean-squared error used in our SNR measurements in Table 1 could be biased for low-photon count comparisons. The mutual information between two images X and Y is computed by $I(X;Y) := \sum_{y\in Y}\sum_{x\in X} p(x,y)\log_2\left(\frac{p(x,y)}{p(x)p(y)}\right)$ where $p(x)$ and $p(y)$ are the probability mass functions of X and Y and $p(x,y)$ is the joint probability mass function of X and Y. As our deconvolved images are represented in floating point number, we computed their probability mass functions, or histograms, with equally-spaced 50 bins ranging from their minimum value to their maximum value. Note that the regularization parameters in each test here were chosen to maximize the mutual information in a manner analogous to how we tuned them to maximize SNR in Table 1.

Object	Number of maximum photoelectrons	Noise model (no regularizer)			3D Regularizer				
		Gaussian	Poisson	S-Poisson	ℓ_2	Laplacian	ℓ_1	TV	FH
Mitochondria	1000	0.095	0.347	0.348	0.400	0.457	0.425	0.470	**0.491**
	500	0.070	0.283	0.284	0.382	0.449	0.398	0.446	**0.466**
	100	0.037	0.151	0.154	0.331	0.406	0.338	0.389	**0.410**
	50	0.029	0.104	0.109	0.301	0.376	0.306	0.360	**0.380**
	10	0.014	0.027	0.035	0.194	0.264	0.191	0.275	**0.292**
Actin	1000	0.057	0.247	0.247	0.262	0.386	0.233	0.387	**0.416**
	500	0.038	0.178	0.179	0.233	0.368	0.221	0.346	**0.376**
	100	0.017	0.071	0.072	0.168	**0.292**	0.176	0.260	0.291
	50	0.012	0.045	0.045	0.155	0.251	0.146	0.235	**0.256**
	10	0.005	0.011	0.013	0.080	0.121	0.085	0.167	**0.167**
Nucleus	1000	0.098	0.334	0.335	0.405	0.375	0.404	0.429	**0.443**
	500	0.074	0.300	0.301	0.392	0.390	0.399	0.422	**0.437**
	100	0.040	0.203	0.208	0.355	**0.413**	0.374	0.399	**0.413**
	50	0.030	0.155	0.163	0.335	**0.410**	0.351	0.385	0.397
	10	0.014	0.048	0.061	0.261	0.337	0.253	0.330	**0.343**

Supplementary Table S3. Mutual information comparison of different noise models and regularization methods. The performance of various noise models and regularizers are compared at different noise levels on simulated fluorescence images. The resulting mutual information are averaged over ten noise realizations. The center columns of the table show a comparison of different noise models (Gaussian, Poisson, and shifted-Poisson) where deconvolution is performed with the non-negativity constraint but without any regularizers. On the right side of the table the shifted-Poisson likelihood function is paired with different regularization methods. The mutual information between the original image and the deconvolved image is computed by constructing histograms with 50 bins.

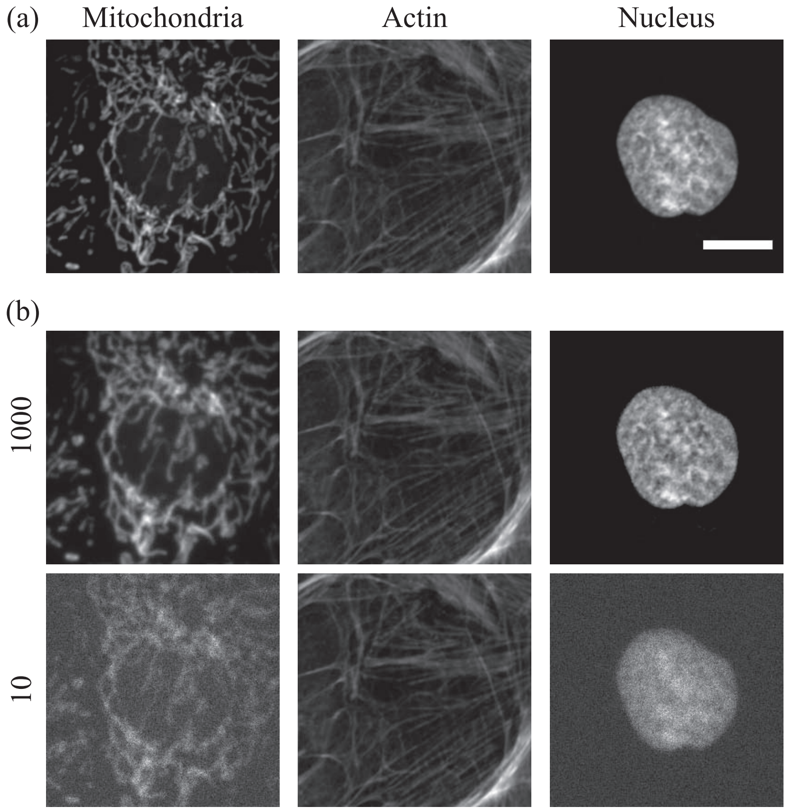

Supplementary Figure S2. (a) Slices from focal stacks ($256 \times 256 \times 22$ voxels) that were intentionally collected at very high SNR and preprocessed so that they could serve as a ground truth data set for noise analysis (see Methods). (b) Simulated Poisson and Gaussian noise was added to the ground truth images. This enabled us to characterize the performance of our deconvolution algorithm at different SNR levels. The numbers on the left side of the figure represent the maximum photoelectron number contained in voxels in each test volume (which is proportional to its noise level). The scale bar has a length of $5\,\mu\text{m}$.

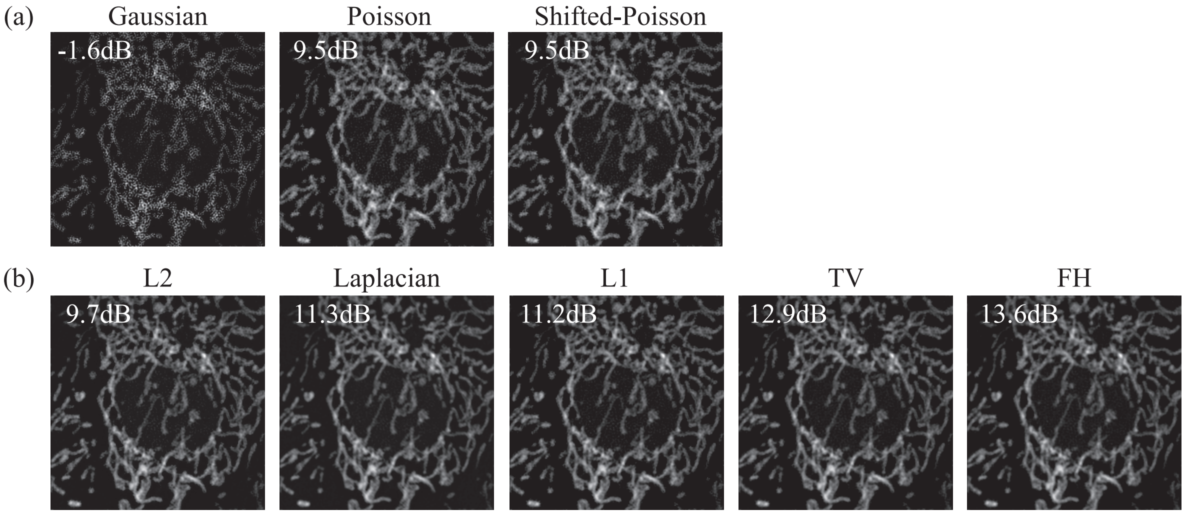

Supplementary Figure S3. Deconvolution of a high-SNR simulated widefield microscopy focal stack (mitochondria) whose maximum photoelectron count is set to 1000. The results show the seventh z slice of the volume after deconvolution. The numbers in the upper-left corner show the SNR of the deconvolved images, which also appear in Table 1 of the main manuscript. The scale bar has a length of $5\,\mu\text{m}$.

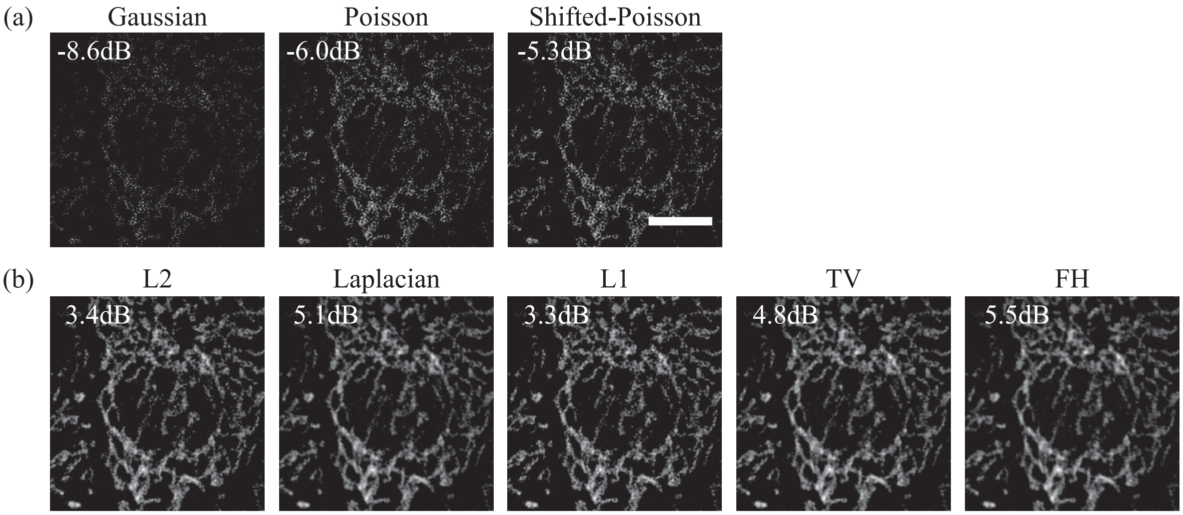

Supplementary Figure S4. Deconvolution of a low-SNR simulated widefield microscopy image (mitochondria), whose maximum photoelectron count is set to 10. The results show the seventh slice of the volume. The numbers in the upper-left corner show the SNR of the deconvolved images, which also appear in Table 1 of the main manuscript. The scale bar has a length of $5\,\mu\mathrm{m}$.

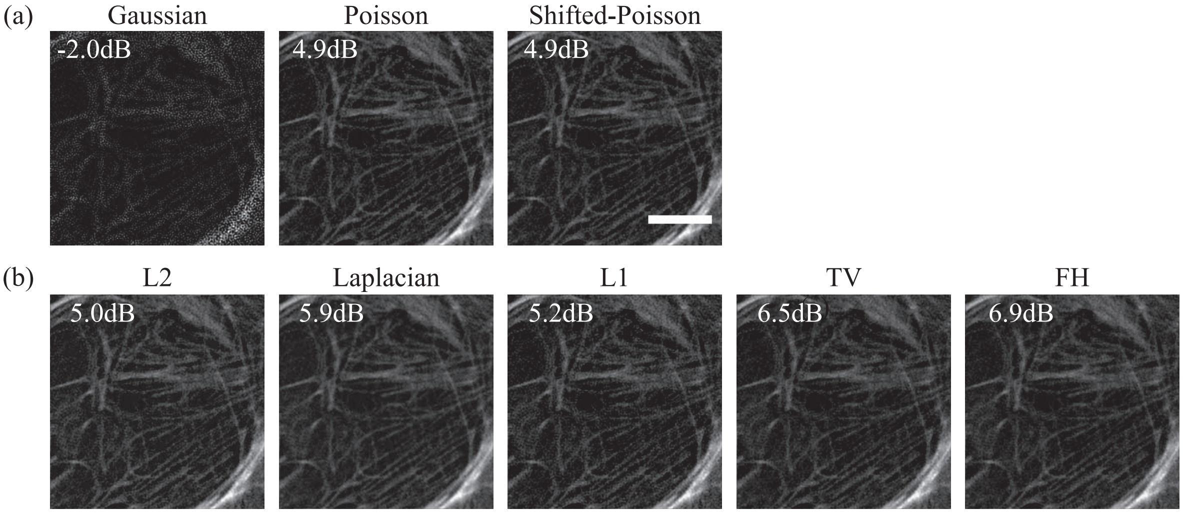

Supplementary Figure S5. Deconvolution of a high-SNR simulated widefield microscopy image (actin), whose maximum photoelectron count is set to 1000. The results show the seventh slice of the volume. The numbers in the upper-left corner show the SNR of the deconvolved images, which also appear in Table 1 of the main manuscript. The scale bar has a length of $5\,\mu\mathrm{m}$.

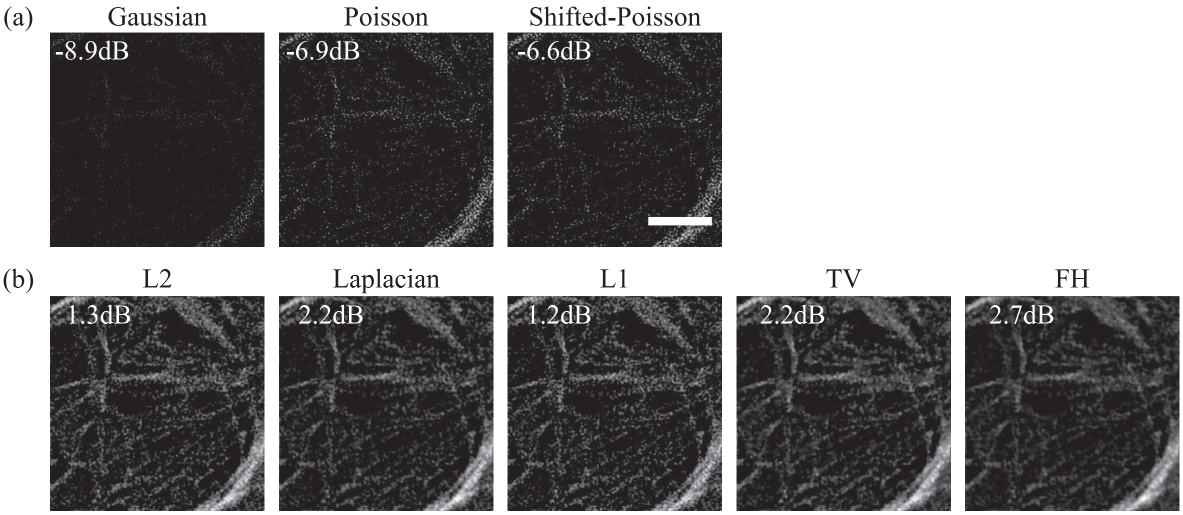

Supplementary Figure S6. Deconvolution of a low-SNR simulated widefield microscopy image (actin), whose maximum photoelectron count is set to 10. The results show the seventh slice of the volume. The numbers in the upper-left corner show the SNR of the deconvolved images, which also appear in Table 1 of the main manuscript. The scale bar has a length of $5\,\mu\mathrm{m}$.

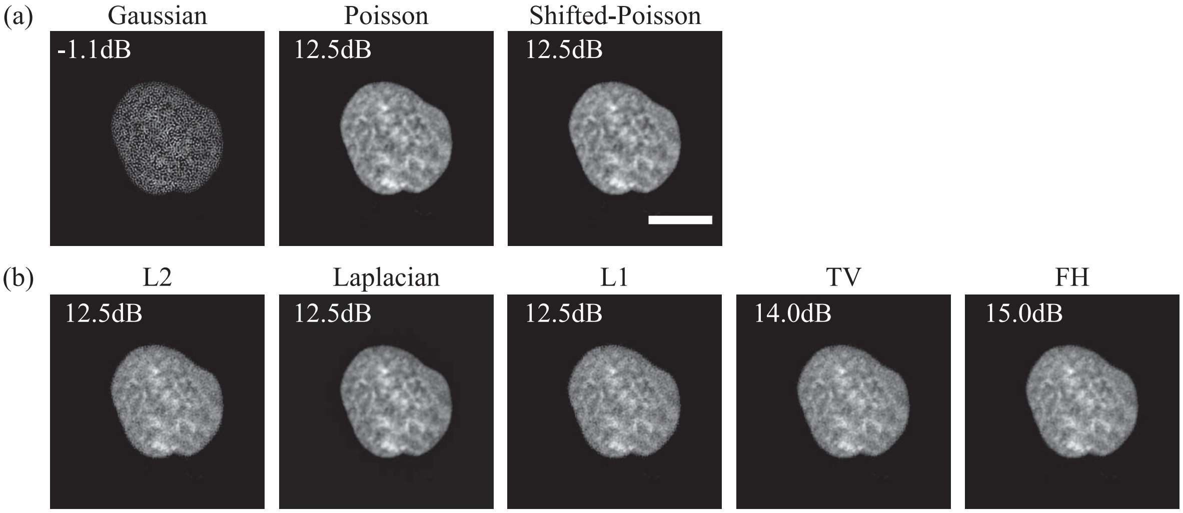

Supplementary Figure S7. Deconvolution of a high-SNR simulated widefield microscopy image (nucleus), whose maximum photoelectron count is set to 1000. The results show the seventh slice of the volume. The numbers in the upper-left corner show the SNR of the deconvolved images, which also appear in Table 1 of the main manuscript. The scale bar has a length of $5\,\mu\mathrm{m}$.

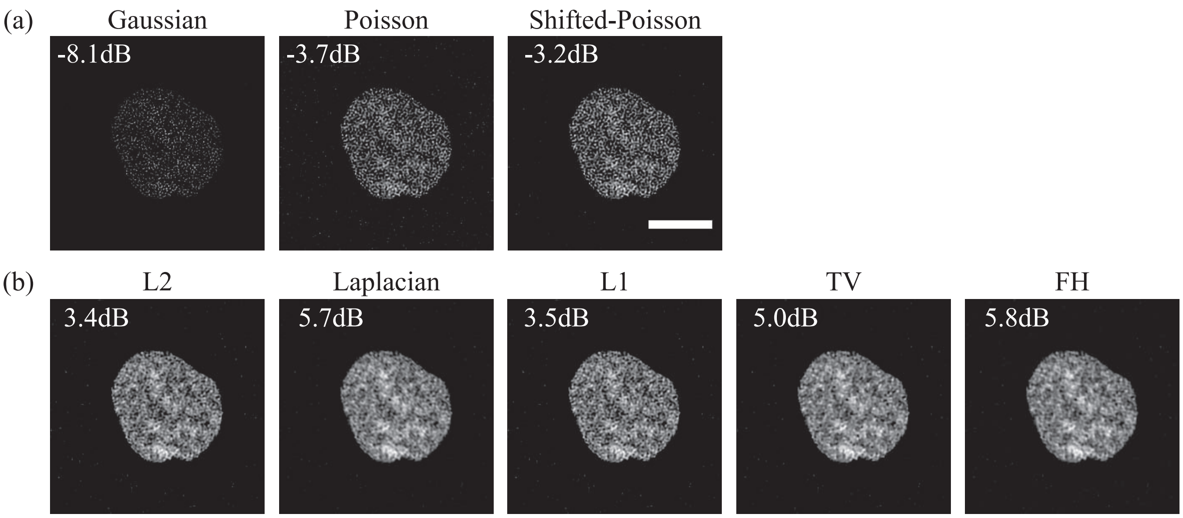

Supplementary Figure S8. Deconvolution of a low-SNR simulated widefield microscopy image (nucleus), whose maximum photoelectron count is set to 10. The results show the seventh slice of the volume. The numbers in the upper-left corner show the SNR of the deconvolved images, which also appear in Table 1 of the main manuscript. The scale bar has a length of $5\,\mu\mathrm{m}$.

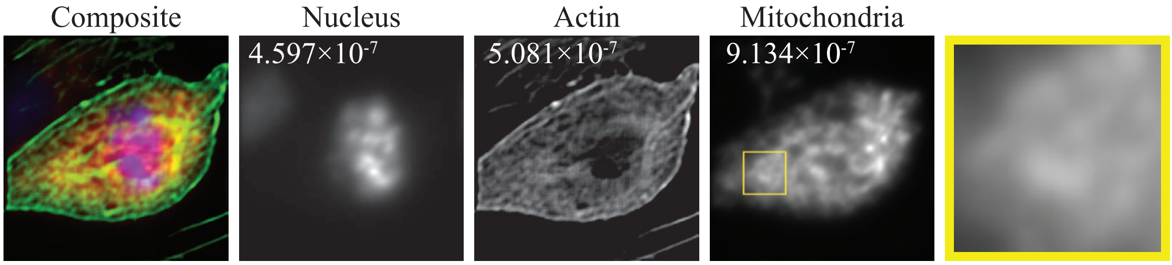

Supplementary Figure S9. Deconvolution of the fixed cells with ER-Decon 2. The regularization parameters were selected from the NMSE analysis. As shown here, the regularization parameter that minimized the NMSE resulted in a blurry image. Thus, we hand-tuned a different regularization parameter that produced a set of visually-pleasant images, and we used this hand-tuned parameter to generate ER-Decon 2 results for Figure 2.

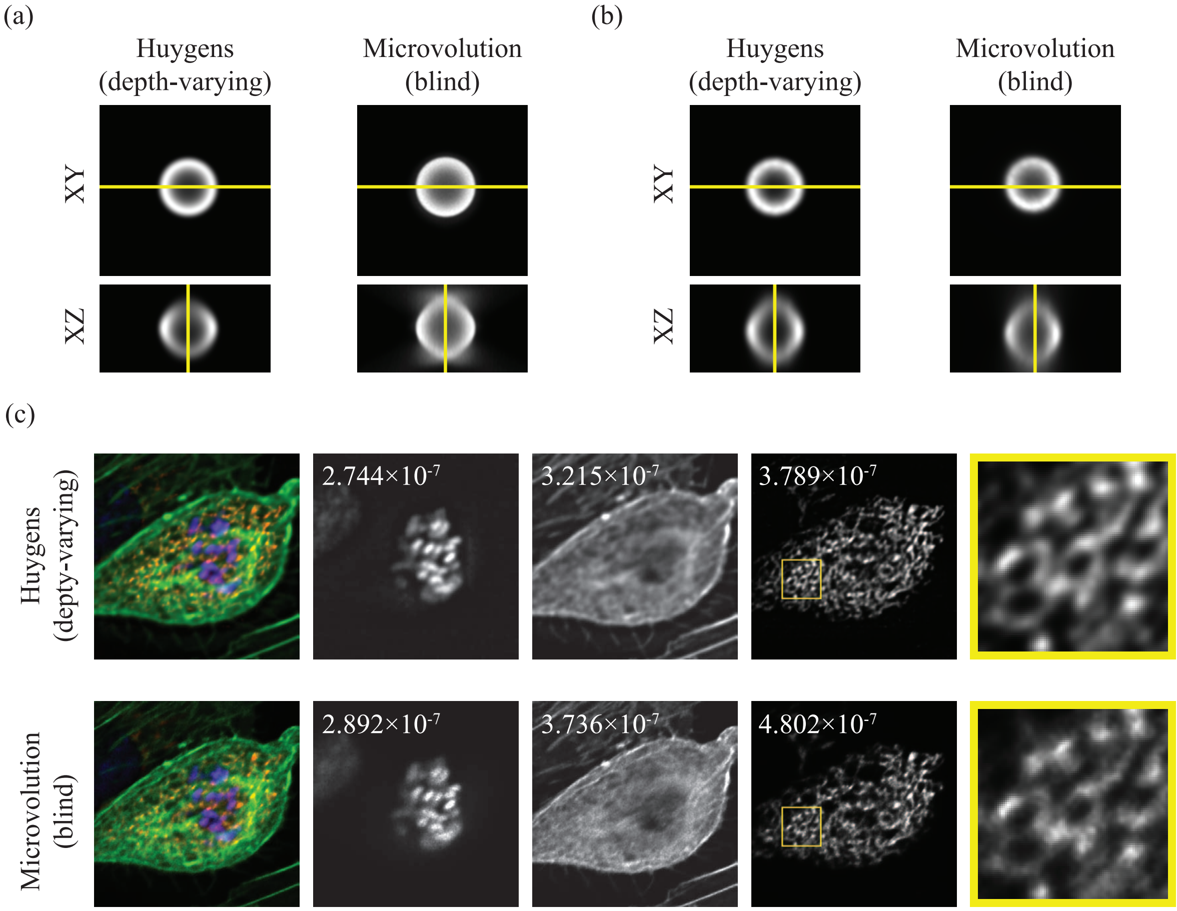

Supplementary Figure S10. Deconvolution of the bead and the fixed cell when using advanced deconvolution modes in Huygens and Microvolution. In this figure, we study the depth-varying PSF mode in Huygens and the blind-deconvolution mode in Microvolution. We keep these results separate because they both utilize more advanced PSF models than the methods (including our own) from the main manuscript. All results in the main manuscript use fixed PSF models that do not vary with depth. These results show that the use of depth-dependent or adaptive PSF model is advantageous, and adding this capability to our algorithm would be a good avenue for future work. The runtimes in these tests were 8.3 s for Huygens (27 iterations) and 22.3 s for Microvolution (100 iterations)

H Statistics of the captured images

Quantile	Bead (high-photon counts)	Bead (low-photon counts)	Mitochondria	Actin	Nucleus
1%	122	92	94	98	94
50%	749	100	102	141	101
99%	24245	139	146	379	127

Supplementary Table S4. Statistics of the raw image digital numbers (DN) shown in Figures 1 and 3 (taken prior to dark frame subtraction or conversion to photoelectron counts). The camera gain factors for the conversion from DN to photoelectron number are 0.46 [electron/DN] for the bead dataset and 0.6 [electron/DN] for other datasets. The camera offsets for all experiments are 100 DN. Throughout this manuscript, we normalize the 3D PSF such that each of its xy slices sums to 1.0. This normalization models the photoelectron counts in the estimated 3D volume as being the contribution over the exposure time of each captured 2D image of the focal stack. Due to the fact that all fluorophores in the volume are excited during each capture of the captured focal slices, the photoelectron counts recorded in each image are roughly the same as the total number of photons emitted by the volume up to the quantum efficiency of the sensor and other scaling factors. Each recorded image can be interpreted as a different realization of the same Poisson process. Therefore, the total number of photoelectrons in the recovered volume is significantly lower than the total number of photoelectrons recorded in the entire focal stack.

Supplementary Video S1. Deconvolution results for experimentally captured focal stacks containing a 6 μm hollow microsphere. The top and bottom rows shows the high and low photon counts images, respectively. This video corresponds to the focal stacks visualized in Figure 1.

Supplementary Video S2. A comparison of 3D deconvolution software used to process a low SNR, $512 \times 512 \times 22$ voxel widefield fluorescence focal stack. This video corresponds to the focal stacks visualized in Figure 2.

Supplementary Video S3. Deconvolution results for imaging of live cells expressing histone H2B fused to mClover to visualize chromosome conformation. This 4D-rendered video corresponds to the focal stacks visualized in Figure 4(a).

Supplementary Video S4. Deconvolution results for imaging of live cells expressing histone H2B fused to mClover to visualize chromosome conformation. This 4D-rendered video corresponds to the focal stacks visualized in Figure 4(b).

References

1. Goodman, J. W. *Introduction to Fourier optics* (Roberts and Company Publishers, 2005).
2. Ding, Q., Long, Y., Zhang, X. & Fessler, J. A. Statistical image reconstruction using mixed poisson-gaussian noise model for x-ray ct. *arXiv 1801.09533* (2018).
3. Boyd, S., Parikh, N., Chu, E., Peleato, B. & Eckstein, J. Distributed optimization and statistical learning via the alternating direction method of multipliers. *Found. Trends Mach. Learn.* **3**, 1–122 (2011).
4. Parikh, N. & Boyd, S. P. Proximal algorithms. *Found. Trends Optim.* **1**, 127–239 (2014).
5. Lefkimmiatis, S. & Unser, M. Poisson image reconstruction with Hessian Schatten-norm regularization. *IEEE Trans. Image Process.* **22**, 4314–4327 (2013).
6. Lefkimmiatis, S., Bourquard, A. & Unser, M. Hessian-based norm regularization for image restoration with biomedical applications. *IEEE Trans. Image Process.* **21**, 983–995 (2012).
7. Heide, F. *et al.* FlexISP: A flexible camera image processing framework. *ACM Trans. Graph.* **33**, 231:1–13 (2014).
8. Heide, F. *et al.* ProxImaL: Efficient image optimization using proximal algorithms. *ACM Trans. Graph.* **35**, 84:1–84 (2016).
9. Bezanson, J., Edelman, A., Karpinski, S. & Shah, V. B. Julia: A fresh approach to numerical computing. *SIAM Review* **59**, 65–98 (2017).
10. Yalamanchili, P. *et al.* ArrayFire - A high performance software library for parallel computing with an easy-to-use API (2015). URL https://github.com/arrayfire/arrayfire.
11. Foi, A., Trimeche, M., Katkovnik, V. & Egiazarian, K. Practical Poissonian-Gaussian noise modeling and fitting for single-image raw-data. *IEEE Trans. Image Process.* **17**, 1737–1754 (2008).
12. Viola, P. & Wells III, W. M. Alignment by maximization of mutual information. *Int. J. Comput. Vis.* **24**, 137–154 (1997).
13. Maes, F., Collignon, A., Vandermeulen, D., Marchal, G. & Suetens, P. Multimodality image registration by maximization of mutual information. *IEEE Trans. Med. Imaging* **16**, 187–198 (1997).
